# Supplementary material for: A Critical View of the Application of the APEX Software (Aqueous Photochemistry of Environmentally-Occurring Xenobiotics) to Predict Photoreaction Kinetics in Surface Freshwaters
Source: Molecules. 2019 Dec 18;25(1):9. doi: 10.3390/molecules25010009 (PMC7017383; doi:10.3390/molecules25010009)
Supplement: Supplementary file 1 [file molecules-25-00009-s001.zip › 3.2.4_gcc-4.4.0.zip!/3.2.4_gcc-4.4.0/3.2.4_gcc-4.4.0/doc/octave/html/interpreter/Signal-Processing.html]

Signal Processing - Untitled


Next: Image Processing,
Previous: Geometry,
Up: Top


---

## 30 Signal Processing

This chapter describes the signal processing and fast Fourier
transform functions available in Octave. Fast Fourier transforms are
computed with the fftw or fftpack libraries depending on how
Octave is built.

— Function File: **detrend** (x, p)  
> If x is a vector, `detrend (`x`,` p`)` removes the
> best fit of a polynomial of order p from the data x.
>
> If x is a matrix, `detrend (`x`,` p`)` does the same
> for each column in x.
>
> The second argument is optional. If it is not specified, a value of 1
> is assumed. This corresponds to removing a linear trend.

— Loadable Function: **fft** (a, n, dim)  
> Compute the FFT of a using subroutines from
> fftw. The FFT is calculated along the first non-singleton dimension of the
> array. Thus if a is a matrix, `fft (`a`)` computes the
> FFT for each column of a.
>
> If called with two arguments, n is expected to be an integer
> specifying the number of elements of a to use, or an empty
> matrix to specify that its value should be ignored. If n is
> larger than the dimension along which the FFT is calculated, then
> a is resized and padded with zeros. Otherwise, if n is
> smaller than the dimension along which the FFT is calculated, then
> a is truncated.
>
> If called with three arguments, dim is an integer specifying the
> dimension of the matrix along which the FFT is performed
>
>
>
> **See also:** ifft, fft2, fftn, fftw.

Octave uses the fftw libraries to perform FFT computations. When Octave
starts up and initializes the fftw libraries, they read a system wide
file (on a Unix system, it is typically /etc/fftw/wisdom) that
contains information useful to speed up FFT computations. This
information is called the *wisdom*. The system-wide file allows
wisdom to be shared between all applications using the fftw libraries.

Use the `fftw` function to generate and save wisdom. Using the
utilities provided together with the fftw libraries
(fftw-wisdom on Unix systems), you can even add wisdom
generated by Octave to the system-wide wisdom file.

— Loadable Function: method = **fftw** ('planner')  
— Loadable Function: **fftw** ('planner', method)  
— Loadable Function: wisdom = **fftw** ('dwisdom')  
— Loadable Function: wisdom = **fftw** ('dwisdom', wisdom)  
> Manage fftw wisdom data. Wisdom data can be used to significantly
> accelerate the calculation of the FFTs but implies an initial cost
> in its calculation. When the fftw libraries are initialized, they read
> a system wide wisdom file (typically in /etc/fftw/wisdom), allowing wisdom
> to be shared between applications other than Octave. Alternatively, the
> `fftw` function can be used to import wisdom. For example
>
> ```
>           wisdom = fftw ('dwisdom')
> ```
>
> will save the existing wisdom used by Octave to the string wisdom.
> This string can then be saved to a file and restored using the `save`
> and `load` commands respectively. This existing wisdom can be reimported
> as follows
>
> ```
>           fftw ('dwisdom', wisdom)
> ```
>
> If wisdom is an empty matrix, then the wisdom used is cleared.
>
> During the calculation of Fourier transforms further wisdom is generated.
> The fashion in which this wisdom is generated is equally controlled by
> the `fftw` function. There are five different manners in which the
> wisdom can be treated, these being
>
> 'estimate': This specifies that no run-time measurement of the optimal means of calculating a particular is performed, and a simple heuristic is used to pick a (probably sub-optimal) plan. The advantage of this method is that there is little or no overhead in the generation of the plan, which is appropriate for a Fourier transform that will be calculated once. 'measure': In this case a range of algorithms to perform the transform is considered and the best is selected based on their execution time. 'patient': This is like 'measure', but a wider range of algorithms is considered. 'exhaustive': This is like 'measure', but all possible algorithms that may be used to treat the transform are considered. 'hybrid': As run-time measurement of the algorithm can be expensive, this is a compromise where 'measure' is used for transforms up to the size of 8192 and beyond that the 'estimate' method is used.
>
> The default method is 'estimate', and the method currently being used can
> be probed with
>
> ```
>           method = fftw ('planner')
> ```
>
> and the method used can be set using
>
> ```
>           fftw ('planner', method)
> ```
>
> Note that calculated wisdom will be lost when restarting Octave. However,
> the wisdom data can be reloaded if it is saved to a file as described
> above. Saved wisdom files should not be used on different platforms since
> they will not be efficient and the point of calculating the wisdom is lost.
>
>
>
> **See also:** fft, ifft, fft2, ifft2, fftn, ifftn.

— Loadable Function: **ifft** (a, n, dim)  
> Compute the inverse FFT of a using subroutines from
> fftw. The inverse FFT is calculated along the first non-singleton dimension
> of the array. Thus if a is a matrix, `fft (`a`)` computes
> the inverse FFT for each column of a.
>
> If called with two arguments, n is expected to be an integer
> specifying the number of elements of a to use, or an empty
> matrix to specify that its value should be ignored. If n is
> larger than the dimension along which the inverse FFT is calculated, then
> a is resized and padded with zeros. Otherwise, ifn is
> smaller than the dimension along which the inverse FFT is calculated,
> then a is truncated.
>
> If called with three arguments, dim is an integer specifying the
> dimension of the matrix along which the inverse FFT is performed
>
>
>
> **See also:** fft, ifft2, ifftn, fftw.

— Loadable Function: **fft2** (a, n, m)  
> Compute the two-dimensional FFT of a using subroutines from
> fftw. The optional arguments n and m may be used specify the
> number of rows and columns of a to use. If either of these is
> larger than the size of a, a is resized and padded with
> zeros.
>
> If a is a multi-dimensional matrix, each two-dimensional sub-matrix
> of a is treated separately
>
>
>
> **See also:** ifft2, fft, fftn, fftw.

— Loadable Function: **fft2** (a, n, m)  
> Compute the inverse two-dimensional FFT of a using subroutines from
> fftw. The optional arguments n and m may be used specify the
> number of rows and columns of a to use. If either of these is
> larger than the size of a, a is resized and padded with
> zeros.
>
> If a is a multi-dimensional matrix, each two-dimensional sub-matrix
> of a is treated separately
>
>
>
> **See also:** fft2, ifft, ifftn, fftw.

— Loadable Function: **fftn** (a, size)  
> Compute the N-dimensional FFT of a using subroutines from
> fftw. The optional vector argument size may be used specify the
> dimensions of the array to be used. If an element of size is
> smaller than the corresponding dimension, then the dimension is
> truncated prior to performing the FFT. Otherwise if an element
> of size is larger than the corresponding dimension a
> is resized and padded with zeros.
>
>
>
> **See also:** ifftn, fft, fft2, fftw.

— Loadable Function: **ifftn** (a, size)  
> Compute the inverse N-dimensional FFT of a using subroutines from
> fftw. The optional vector argument size may be used specify the
> dimensions of the array to be used. If an element of size is
> smaller than the corresponding dimension, then the dimension is
> truncated prior to performing the inverse FFT. Otherwise if an element
> of size is larger than the corresponding dimension a
> is resized and padded with zeros.
>
>
>
> **See also:** fftn, ifft, ifft2, fftw.

— Function File: **fftconv** (a, b, n)  
> Return the convolution of the vectors a and b, as a vector
> with length equal to the `length (a) + length (b) - 1`. If a
> and b are the coefficient vectors of two polynomials, the returned
> value is the coefficient vector of the product polynomial.
>
> The computation uses the FFT by calling the function `fftfilt`. If
> the optional argument n is specified, an N-point FFT is used.

— Function File: **fftfilt** (b, x, n)  
> With two arguments, `fftfilt` filters x with the FIR filter
> b using the FFT.
>
> Given the optional third argument, n, `fftfilt` uses the
> overlap-add method to filter x with b using an N-point FFT.
>
> If x is a matrix, filter each column of the matrix.

— Loadable Function: y = **filter** (b, a, x)  
— Loadable Function: [y, sf] = **filter** (b, a, x, si)  
— Loadable Function: [y, sf] = **filter** (b, a, x, [], dim)  
— Loadable Function: [y, sf] = **filter** (b, a, x, si, dim)  
> Return the solution to the following linear, time-invariant difference
> equation:
>
> ```
>              N                   M
>             SUM a(k+1) y(n-k) = SUM b(k+1) x(n-k)      for 1<=n<=length(x)
>             k=0                 k=0
> ```
>
> where
> N=length(a)-1 and M=length(b)-1.
> over the first non-singleton dimension of x or over dim if
> supplied. An equivalent form of this equation is:
>
> ```
>                       N                   M
>             y(n) = - SUM c(k+1) y(n-k) + SUM d(k+1) x(n-k)  for 1<=n<=length(x)
>                      k=1                 k=0
> ```
>
> where
> c = a/a(1) and d = b/a(1).
>
> If the fourth argument si is provided, it is taken as the
> initial state of the system and the final state is returned as
> sf. The state vector is a column vector whose length is
> equal to the length of the longest coefficient vector minus one.
> If si is not supplied, the initial state vector is set to all
> zeros.
>
> In terms of the z-transform, y is the result of passing the discrete-
> time signal x through a system characterized by the following rational
> system function:
>
> ```
>                        M
>                       SUM d(k+1) z^(-k)
>                       k=0
>             H(z) = ----------------------
>                          N
>                     1 + SUM c(k+1) z^(-k)
>                         k=1
> ```

— Function File: y = **filter2** (b, x)  
— Function File: y = **filter2** (b, x, shape)  
> Apply the 2-D FIR filter b to x. If the argument
> shape is specified, return an array of the desired shape.
> Possible values are:
>
> 'full': pad x with zeros on all sides before filtering. 'same': unpadded x (default) 'valid': trim x after filtering so edge effects are no included.
>
> Note this is just a variation on convolution, with the parameters
> reversed and b rotated 180 degrees.
>
>
>
> **See also:** conv2.

— Function File: [h, w] = **freqz** (b, a, n, "whole")  
> Return the complex frequency response h of the rational IIR filter
> whose numerator and denominator coefficients are b and a,
> respectively. The response is evaluated at n angular frequencies
> between 0 and
> 2\*pi.
>
> The output value w is a vector of the frequencies.
>
> If the fourth argument is omitted, the response is evaluated at
> frequencies between 0 and
> pi.
>
> If n is omitted, a value of 512 is assumed.
>
> If a is omitted, the denominator is assumed to be 1 (this
> corresponds to a simple FIR filter).
>
> For fastest computation, n should factor into a small number of
> small primes.
> — Function File: h = **freqz** (b, a, w)  
> > Evaluate the response at the specific frequencies in the vector w.
> > The values for w are measured in radians.
> > — Function File: [...] = **freqz** (..., Fs)  
> > > Return frequencies in Hz instead of radians assuming a sampling rate
> > > Fs. If you are evaluating the response at specific frequencies
> > > w, those frequencies should be requested in Hz rather than radians.
> > > — Function File: **freqz** (...)  
> > > > Plot the pass band, stop band and phase response of h rather
> > > > than returning them.

— Function File: **freqz\_plot** (w, h)  
> Plot the pass band, stop band and phase response of h.

— Function File: **sinc** (x)  
> Return
> sin(pi\*x)/(pi\*x).

— Function File: b = **unwrap** (a, tol, dim)  
> Unwrap radian phases by adding multiples of 2\*pi as appropriate to
> remove jumps greater than tol. tol defaults to pi.
>
> Unwrap will unwrap along the first non-singleton dimension of
> a, unless the optional argument dim is given, in
> which case the data will be unwrapped along this dimension


— Function File: [a, b] = **arch\_fit** (y, x, p, iter, gamma, a0, b0)  
> Fit an ARCH regression model to the time series y using the
> scoring algorithm in Engle's original ARCH paper. The model is
>
> ```
>           y(t) = b(1) * x(t,1) + ... + b(k) * x(t,k) + e(t),
>           h(t) = a(1) + a(2) * e(t-1)^2 + ... + a(p+1) * e(t-p)^2
> ```
>
> in which e(t) is N(0, h(t)), given a time-series vector
> y up to time t-1 and a matrix of (ordinary) regressors
> x up to t. The order of the regression of the residual
> variance is specified by p.
>
> If invoked as `arch_fit (`y`,` k`,` p`)` with a
> positive integer k, fit an ARCH(k, p) process,
> i.e., do the above with the t-th row of x given by
>
> ```
>           [1, y(t-1), ..., y(t-k)]
> ```
>
> Optionally, one can specify the number of iterations iter, the
> updating factor gamma, and initial values a0 and
> b0 for the scoring algorithm.

— Function File: **arch\_rnd** (a, b, t)  
> Simulate an ARCH sequence of length t with AR
> coefficients b and CH coefficients a. I.e., the result
> y(t) follows the model
>
> ```
>           y(t) = b(1) + b(2) * y(t-1) + ... + b(lb) * y(t-lb+1) + e(t),
> ```
>
> where e(t), given y up to time t-1, is
> N(0, h(t)), with
>
> ```
>           h(t) = a(1) + a(2) * e(t-1)^2 + ... + a(la) * e(t-la+1)^2
> ```

— Function File: [pval, lm] = **arch\_test** (y, x, p)  
> For a linear regression model
>
> ```
>           y = x * b + e
> ```
>
> perform a Lagrange Multiplier (LM) test of the null hypothesis of no
> conditional heteroscedascity against the alternative of CH(p).
>
> I.e., the model is
>
> ```
>           y(t) = b(1) * x(t,1) + ... + b(k) * x(t,k) + e(t),
> ```
>
> given y up to t-1 and x up to t,
> e(t) is N(0, h(t)) with
>
> ```
>           h(t) = v + a(1) * e(t-1)^2 + ... + a(p) * e(t-p)^2,
> ```
>
> and the null is a(1) == ... == a(p) == 0.
>
> If the second argument is a scalar integer, k, perform the same
> test in a linear autoregression model of order k, i.e., with
>
> ```
>           [1, y(t-1), ..., y(t-k)]
> ```
>
> as the t-th row of x.
>
> Under the null, LM approximately has a chisquare distribution with
> p degrees of freedom and pval is the p-value (1
> minus the CDF of this distribution at LM) of the test.
>
> If no output argument is given, the p-value is displayed.

— Function File: **arma\_rnd** (a, b, v, t, n)  
> Return a simulation of the ARMA model
>
> ```
>           x(n) = a(1) * x(n-1) + ... + a(k) * x(n-k)
>                + e(n) + b(1) * e(n-1) + ... + b(l) * e(n-l)
> ```
>
> in which k is the length of vector a, l is the
> length of vector b and e is Gaussian white noise with
> variance v. The function returns a vector of length t.
>
> The optional parameter n gives the number of dummy
> x(i) used for initialization, i.e., a sequence of length
> t+n is generated and x(n+1:t+n)
> is returned. If n is omitted, n = 100 is used.

— Function File: **autocor** (x, h)  
> Return the autocorrelations from lag 0 to h of vector x.
> If h is omitted, all autocorrelations are computed.
> If x is a matrix, the autocorrelations of each column are
> computed.

— Function File: **autocov** (x, h)  
> Return the autocovariances from lag 0 to h of vector x.
> If h is omitted, all autocovariances are computed.
> If x is a matrix, the autocovariances of each column are
> computed.

— Function File: **autoreg\_matrix** (y, k)  
> Given a time series (vector) y, return a matrix with ones in the
> first column and the first k lagged values of y in the
> other columns. I.e., for t > k, `[1,` y`(`t`-1), ...,` y`(`t`-`k`)]` is the t-th row
> of the result. The resulting matrix may be used as a regressor matrix
> in autoregressions.

— Function File: **bartlett** (m)  
> Return the filter coefficients of a Bartlett (triangular) window of
> length m.
>
> For a definition of the Bartlett window, see e.g., A. V. Oppenheim &
> R. W. Schafer, Discrete-Time Signal Processing.

— Function File: **blackman** (m)  
> Return the filter coefficients of a Blackman window of length m.
>
> For a definition of the Blackman window, see e.g., A. V. Oppenheim &
> R. W. Schafer, Discrete-Time Signal Processing.

— Function File: [d, dd] = **diffpara** (x, a, b)  
> Return the estimator d for the differencing parameter of an
> integrated time series.
>
> The frequencies from [2\*pi\*a/t, 2\*pi\*b/T] are used for the
> estimation. If b is omitted, the interval
> [2\*pi/T, 2\*pi\*a/T] is used. If both b and a are
> omitted then a = 0.5 \* sqrt (T) and b = 1.5 \* sqrt (T)
> is used, where T is the sample size. If x is a matrix,
> the differencing parameter of each column is estimated.
>
> The estimators for all frequencies in the intervals
> described above is returned in dd. The value of d is
> simply the mean of dd.
>
> Reference: Brockwell, Peter J. & Davis, Richard A. Time Series:
> Theory and Methods Springer 1987.

— Function File: **durbinlevinson** (c, oldphi, oldv)  
> Perform one step of the Durbin-Levinson algorithm.
>
> The vector c specifies the autocovariances `[gamma_0, ...,
> gamma_t]` from lag 0 to t, oldphi specifies the
> coefficients based on c(t-1) and oldv specifies the
> corresponding error.
>
> If oldphi and oldv are omitted, all steps from 1 to
> t of the algorithm are performed.

— Function File: **fftshift** (v)  
— Function File: **fftshift** (v, dim)  
> Perform a shift of the vector v, for use with the `fft`
> and `ifft` functions, in order the move the frequency 0 to the
> center of the vector or matrix.
>
> If v is a vector of N elements corresponding to N
> time samples spaced of Dt each, then `fftshift (fft
> (`v`))` corresponds to frequencies
>
> ```
>           f = ((1:N) - ceil(N/2)) / N / Dt
> ```
>
> If v is a matrix, the same holds for rows and columns. If
> v is an array, then the same holds along each dimension.
>
> The optional dim argument can be used to limit the dimension
> along which the permutation occurs.

— Function File: **ifftshift** (v)  
— Function File: **ifftshift** (v, dim)  
> Undo the action of the `fftshift` function. For even length
> v, `fftshift` is its own inverse, but odd lengths differ
> slightly.

— Function File: **fractdiff** (x, d)  
> Compute the fractional differences (1-L)^d x where L
> denotes the lag-operator and d is greater than -1.

— Function File: **hamming** (m)  
> Return the filter coefficients of a Hamming window of length m.
>
> For a definition of the Hamming window, see e.g., A. V. Oppenheim &
> R. W. Schafer, Discrete-Time Signal Processing.

— Function File: **hanning** (m)  
> Return the filter coefficients of a Hanning window of length m.
>
> For a definition of this window type, see e.g., A. V. Oppenheim &
> R. W. Schafer, Discrete-Time Signal Processing.

— Function File: **hurst** (x)  
> Estimate the Hurst parameter of sample x via the rescaled range
> statistic. If x is a matrix, the parameter is estimated for
> every single column.

— Function File: pp = **pchip** (x, y)  
— Function File: yi = **pchip** (x, y, xi)  
> Piecewise Cubic Hermite interpolating polynomial. Called with two
> arguments, the piece-wise polynomial pp is returned, that may
> later be used with `ppval` to evaluate the polynomial at
> specific points.
>
> The variable x must be a strictly monotonic vector (either
> increasing or decreasing). While y can be either a vector or
> array. In the case where y is a vector, it must have a length
> of n. If y is an array, then the size of y must
> have the form
> `[`s1`,` s2`, ...,` sk`,` n`]`
> The array is then reshaped internally to a matrix where the leading
> dimension is given by
> s1 `*` s2 `* ... *` sk
> and each row in this matrix is then treated separately. Note that this
> is exactly the opposite treatment than `interp1` and is done
> for compatibility.
>
> Called with a third input argument, `pchip` evaluates the
> piece-wise polynomial at the points xi. There is an equivalence
> between `ppval (pchip (`x`,` y`),` xi`)` and
> `pchip (`x`,` y`,` xi`)`.
>
>
>
> **See also:** spline, ppval, mkpp, unmkpp.

— Function File: **periodogram** (x)  
> For a data matrix x from a sample of size n, return the
> periodogram.

— Function File: **rectangle\_lw** (n, b)  
> Rectangular lag window. Subfunction used for spectral density
> estimation.

— Function File: **rectangle\_sw** (n, b)  
> Rectangular spectral window. Subfunction used for spectral density
> estimation.

— Function File: **sinetone** (freq, rate, sec, ampl)  
> Return a sinetone of frequency freq with length of sec
> seconds at sampling rate rate and with amplitude ampl.
> The arguments freq and ampl may be vectors of common size.
>
> Defaults are rate = 8000, sec = 1 and ampl = 64.

— Function File: **sinewave** (m, n, d)  
> Return an m-element vector with i-th element given by
> `sin (2 * pi * (`i`+`d`-1) /` n`)`.
>
> The default value for d is 0 and the default value for n
> is m.

— Function File: **spectral\_adf** (c, win, b)  
> Return the spectral density estimator given a vector of
> autocovariances c, window name win, and bandwidth,
> b.
>
> The window name, e.g., `"triangle"` or `"rectangle"` is
> used to search for a function called win`_sw`.
>
> If win is omitted, the triangle window is used. If b is
> omitted, `1 / sqrt (length (`x`))` is used.

— Function File: **spectral\_xdf** (x, win, b)  
> Return the spectral density estimator given a data vector x,
> window name win, and bandwidth, b.
>
> The window name, e.g., `"triangle"` or `"rectangle"` is
> used to search for a function called win`_sw`.
>
> If win is omitted, the triangle window is used. If b is
> omitted, `1 / sqrt (length (`x`))` is used.

— Function File: **spencer** (x)  
> Return Spencer's 15 point moving average of every single column of
> x.

— Function File: [y, c] = **stft** (x, win\_size, inc, num\_coef, w\_type)  
> Compute the short-time Fourier transform of the vector x with
> num\_coef coefficients by applying a window of win\_size data
> points and an increment of inc points.
>
> Before computing the Fourier transform, one of the following windows
> is applied:
>
> hanning: w\_type = 1 hamming: w\_type = 2 rectangle: w\_type = 3
>
> The window names can be passed as strings or by the w\_type number.
>
> If not all arguments are specified, the following defaults are used:
> win\_size = 80, inc = 24, num\_coef = 64, and
> w\_type = 1.
>
> y `= stft (`x`, ...)` returns the absolute values
> of the Fourier coefficients according to the num\_coef positive
> frequencies.
>
> `[`y`,` c`] = stft (x, ...)` returns the
> entire STFT-matrix y and a 3-element vector c containing
> the window size, increment, and window type, which is needed by the
> synthesis function.

— Function File: **synthesis** (y, c)  
> Compute a signal from its short-time Fourier transform y and a
> 3-element vector c specifying window size, increment, and
> window type.
>
> The values y and c can be derived by
>
> ```
>           [y, c] = stft (x , ...)
> ```

— Function File: **triangle\_lw** (n, b)  
> Triangular lag window. Subfunction used for spectral density
> estimation.

— Function File: **triangle\_sw** (n, b)  
> Triangular spectral window. Subfunction used for spectral density
> estimation.

— Function File: [a, v] = **yulewalker** (c)  
> Fit an AR (p)-model with Yule-Walker estimates given a vector c
> of autocovariances `[gamma_0, ..., gamma_p]`.
>
> Returns the AR coefficients, a, and the variance of white
> noise, v.
